# Developing an Electrochemically Reversible Switch for Modulating the Optical Signal of Gold Nanoparticles

**DOI:** 10.3390/molecules28176233

**Published:** 2023-08-24

**Authors:** Mengran Tang, Long Zhang, Xiaoxue Song, Long Zhao

**Affiliations:** School of Chemistry and Chemical Engineering, Jiangsu University, Zhenjiang 212013, China; 2222112039@stmail.ujs.edu.cn (M.T.); longzhang@ujs.edu.cn (L.Z.); songxiaoxue@stmail.ujs.edu.cn (X.S.)

**Keywords:** gold nanoparticles, nitroxide radicals, electrochemical switch, optical regulation

## Abstract

Gold nanoparticles (AuNPs) possess remarkable optical properties and electrical conductivity, making them highly relevant in various fields such as medical diagnoses, biological imaging, and electronic sensors. However, the existing methods for modulating the optical properties of AuNPs are often under limitations such as a high cost, the complexity of detection, a narrow range of application settings, and irreversibility. In this study, we propose a novel approach to address these challenges by constructing a reversible electrochemical switch. The switch (ITO-OMAD) involves covalently linking nitroxide radicals and AuNPs (AuNPs-NO•), followed by tethering this nanocomposite to a siloxane-derived indium tin oxide (ITO) electrode. By simply electrochemically oxidizing/reducing the nitroxide units, one is able to reversibly modulate the optical properties of AuNPs at will. The surface morphology and structure of the as-prepared ITO-OMAD electrode were characterized through scanning electron microscopy (SEM) and cyclic voltammetry (CV). SEM imaging confirmed the successful anchoring of AuNPs on the ITO electrode. Electrochemical tests performed in the three-electrode system demonstrated that the local surface plasmon resonance (LSPR) of AuNPs can be reversibly regulated by alternatively imposing ± 0.5V (vs. Ag/AgCl) to the modified electrode. The development of this electrochemical switch presents a novel approach to effectively control the optical properties of AuNPs. The further exploration and utilization of this reversible electrochemical switch could significantly enhance the versatility and practicality of AuNPs in numerous applications.

## 1. Introduction

Gold nanoparticles (AuNPs) have emerged as one of the most promising nanomaterials in the 21st century, attributed to their size-dependent behavior [1], facile surface modification capabilities [2], distinctive optical properties [3], high conductivity [4,5,6,7], and remarkable biocompatibility [8,9,10,11,12]. These exceptional characteristics make AuNPs widely utilized in various fields, including optics [3,13], magnetism [14], electronics [15,16], and medicine [2,17,18,19,20]. Given their excellent optical properties, such as local surface plasmon resonance (LSPR), AuNPs have been the subject of extensive research and have broad prospects in different fields [20,21].

In the pursuit of achieving deliberate control over the optical signal of AuNPs, researchers, both domestic and international, have explored various methods to modulate these properties through variations in their composition [21,22], shape [23,24], size, and surrounding medium [25,26]. Zhu et al. discovered significant changes in the LSPR absorption peak of AuNPs with different particle sizes, observing a gradual red shift as the particle size increased [27]. Similarly, Yuan’s group found distinct variations in the LSPR absorption peaks of AuNPs by altering the protective agent used during synthesis [28]. Leroux and his co-worker successfully developed an electrochemical switch between AuNPs and conductive polymers in active plasma devices, allowing for the precise tuning of the LSPR of AuNPs by adjusting the potential imposed on the conductive polymer electrode [15]. Despite these advancements, the current methods employed to regulate the optical properties of AuNPs suffer from several shortcomings, such as sophisticated preparation processes, complex detection procedures, a limited applicability, and irreversibility.

In recent years, there have been great breakthroughs in the research on developing facile approaches to regulate the optical properties of luminous materials. The Blinco research group conducted pioneering work by designing and synthesizing a series of complexes that combined fluorophores and nitroxyl radicals via covalent bonding. This innovative approach enabled the control of fluorescence quenching and recovery in fluorescent units by chemically oxidizing and reducing radical units. Furthermore, the on–off fluorescence behavior of these complexes could be used to detect changes in the oxygen concentration in vivo and in vitro, as well as the generation and reaction of free radicals [29]. Chittreeya and other researchers extended the application by linking nitroxide radicals with quantum dots. Specifically, they harnessed the stable redox activity of radicals to study and modulate the fluorescence properties of CdSe quantum dots. The modification of luminescent materials using nitroxide radicals provides an effective means to regulate their optical properties by switching the open/closed shell state of free radicals [30]. However, it should be noted that these regulation methods primarily rely on redox chemical reactions to alter the state of the radical species, potentially impacting the detection of the optical properties of luminous materials by the introduction of external chemicals, and taking the introduced substances into consideration is necessary to ensure accurate optical property measurements.

(2,2,6,6-Tetramethylpiperidin-1-yl)oxyl (TEMPO) and its derivatives are well-established nitroxide radicals with a number of applications, including organic synthesis [31,32], spin markers [33,34], and electrode materials [16,35]. Their complexes have also demonstrated favorable electrochemical redox activity in both aqueous and organic media [36,37,38]. Herein, this study focuses on constructing an electrochemical switch, namely AuNPs-NO•, by covalently linking nitroxide radicals to AuNPs. By employing electrochemical techniques, the state of the nitroxide radicals could be adjusted upon changing the imposed biases, thereby enabling precise control over the optical properties of the AuNPs. This method offers the advantage of reversible regulation, presenting a novel and facile approach for modulating the optical properties of AuNPs.

## 2. Results and Discussion

### 2.1. Characterization of Synthetic Materials

**Figure 1:**
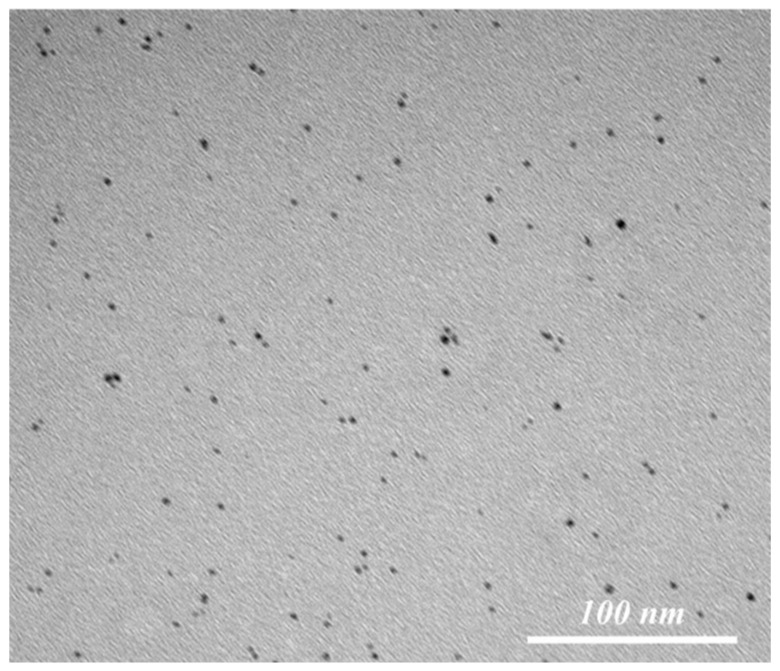
Representative TEM image of AuNPs dispersed in ethanol with an average size of 5 nm.

**Figure 2:**
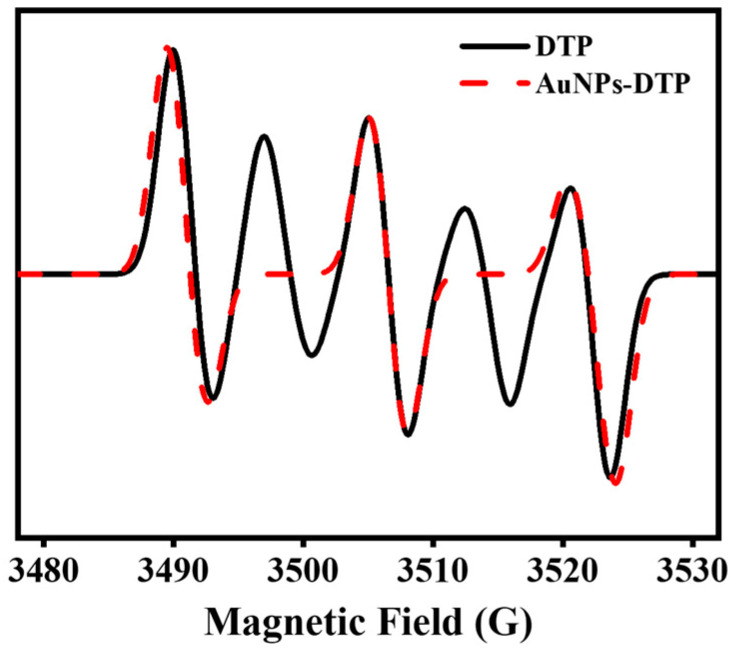
EPR spectra of DTP (black solid line) and AuNPs-DTP (red dashed line) in a toluene solution. EPR instrument settings: 9.85 GHz microwave frequency; 1-milliwatt microwave power; 1-Gauss modulation amplitude; 294 K.

Figure 1 shows the TEM image of solid-dispersed AuNPs in an ethanol solution achieved through the ultrasonication pretreatment. The TEM image illustrates that the synthesized AuNPs exhibit a spherical shape with a uniform dispersion and without agglomeration. The average particle size is approximately 5 nm. In order to study the binding of AuNPs to DTP, EPR tests were performed on both DTP and AuNPs-DTP. Figure 2 displays the initial characteristic EPR spectrum of DTP, where the presence of five wide line spectra indicates the coupling between two free radical centers within the molecule [39]. In contrast, the EPR spectrum of AuNPs-DTP shows the absence of the diradical line, suggesting that the nitroxide radicals, once bound to the surface of the AuNPs, are no longer in close proximity to each other. This result suggests that the disulfide bond within the DTP molecule is cleaved at the center, generating a more stable Au-S bond with the surface of the AuNPs [40,41].

### 2.2. Characterization of the Modified ITO Electrodes

The formation of the ITO-O electrode involves the activation of the ITO-B electrode, resulting in the creation of a molecular layer with a terminal hydroxyl group (–OH) on the surface of the ITO electrode [42]. Upon immersion of the ITO-O electrode in an MPS solution, the trimethoxysilane group on the MPS molecule undergoes condensation with the –OH on the surface of the ITO-O electrode, forming a stable -Si-O-ITO bond. This bond allows for the robust attachment of the MPS monolayer to the ITO surface, while the thiol group (–SH) at the distal end of the MPS remains available for further functionalization [16,42]. Subsequently, the ITO-OM electrode is immersed in a toluene solution of AuNPs-DTP, leading to the binding of the AuNP surface to the sulfhydryl group of MPS through the formation of a strong Au-S bond. This anchoring process securely attaches the AuNPs onto the MPS monolayer on the ITO surface, generating the ITO-OMAD electrode [16].

**Figure 3:**
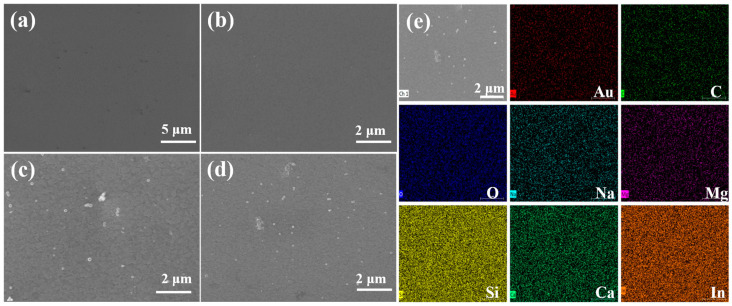
The SEM images of different ITO electrodes (**a**–**d**) and the EDS element maps of ITO electrodes (**e**). (**a**) Bare ITO electrode (ITO-B), (**b**) the hydroxylated ITO electrode condensed with the trimethoxysilane group of MPS to form a thiol-group-terminated ITO electrode (ITO-OM), (**c**) the ITO-OM electrode and AuNPs after being further functionalized by a Au-S bond to produce a AuNP-functionalized ITO electrode (ITO-OMA), (**d**) the ITO-OM electrode and AuNPs-NO• after also being derived by a Au-S bond to generate a AuNPs-NO•-functionalized ITO electrode (ITO-OMAD), and (**e**) EDS mapping spectrum of ITO-OMAD electrode.

Figure 3 presents the surface morphology of the ITO electrode before and after modification, as observed by SEM testing along with the energy spectrum analysis of the ITO-OMAD. It can be noted that the construction of SAM leads to an increasingly rough surface of the ITO electrode (Figure 3a–d). Secondly, the white spots in Figure 3c,d are aggregated AuNPs. Figure 3e shows the energy spectrum analysis diagram of the ITO-OMAD electrode surface, revealing the even distribution of Au on the ITO electrode, with the other elements corresponding to materials present in the ITO substrate.

**Figure 4:**
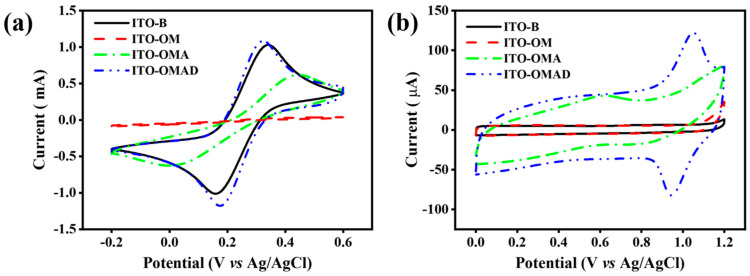
CV diagrams of different ITO electrodes in (**a**) 0.1 M KCl in the presence of 1 mM K_3_[Fe(CN)_6_] and (**b**) acetonitrile in the presence of 0.1 M TBAPF_6_. All CV data were recorded using a scan rate of 1 V/s.

The quality of SAM on the ITO electrode surface and its blocking behavior was evaluated by studying the electron transfer reaction of redox-probe molecules at the electrode/electrolyte interface through CV experiments. In this study, the ITO-B, ITO-OM, ITO-OMA, and ITO-OMAD were analyzed using K_3_[Fe(CN)_6_] as the redox-probe molecules. Figure 4a illustrates the CV curves of the different electrodes in a 0.1 M KCl electrolyte solution of 1 mM K_3_[Fe(CN)_6_] with a scan rate of 1 V/s. It was found that the ITO-B electrode exhibited a well-defined redox peak (black solid line in Figure 4a). This suggests that the electron transfer reaction is predominately controlled by diffusion, demonstrating that electrons can readily transfer on the surface of the ITO-B electrode [43]. The ITO-OM electrode displayed a complete blockade of electron transfer and without a redox peak (red dashed line in Figure 4a). This confirms that the electron transfer on this electrode surface is inhibited. Similarly, the ITO-OMA electrode had an apparent blocking behavior for electron transfer (green dotted/dashed line in Figure 4a), and there was an obvious decrease in the intensity of the redox peaks in comparison to the ITO-B electrode, which indicates that the electron transfer reaction on the electrode surface is partially suppressed. When it comes to the ITO-OMAD electrode, it showed a redox peak of K_3_[Fe(CN)_6_] (blue double dotted/dashed line in Figure 4a), indicating that the surface of the ITO-OMAD electrode also supports electron transfer. Importantly, the specific capacitance of the ITO-OMA electrode is between that of ITO-OM and ITO-B, and the appearance of this small current arises from the attachment of highly conductive AuNPs. This change is due to the formation of a stable Au-S bond between the AuNPs and the -SH at the end of the MPS, which fixes the AuNPs onto the molecular layer, providing conductive sites for electron transfer [43]. In addition, the specific capacitance of the ITO-OMAD electrode is much larger than that of the ITO-OMA electrode and comparable to that of the ITO-B electrode. This can be tentatively ascribed to the fact that the electron transfer is facilitated due to the successful introduction of nitroxide radicals.

The following CV tests were conducted to investigate the presence of a TEMPO redox peak on the ITO-OMAD electrode. Figure 4b (blue double dotted/dashed line) shows the CV curves of different electrodes in acetonitrile containing a supporting electrolyte of 0.1 M TBAPF_6_ at a scan rate of 1 V/s. None of the ITO-O, ITO-OM, or ITO-OMA electrodes present redox peaks, which is because redox-active units do not exist on the surface of these electrodes. In contrast, the CV curve of the ITO-OMAD electrode displays an oxidation peak (+1.1 V) and a reduction peak (+0.9 V), which are the characteristic peaks associated with the TEMPO redox reaction [40]. Thus, it can be concluded that the surface of the ITO-OMAD electrode forms a nitrogen–oxygen free base layer.

### 2.3. Electrochemical Reversible Regulation of AuNP Optical Properties

**Figure 5:**
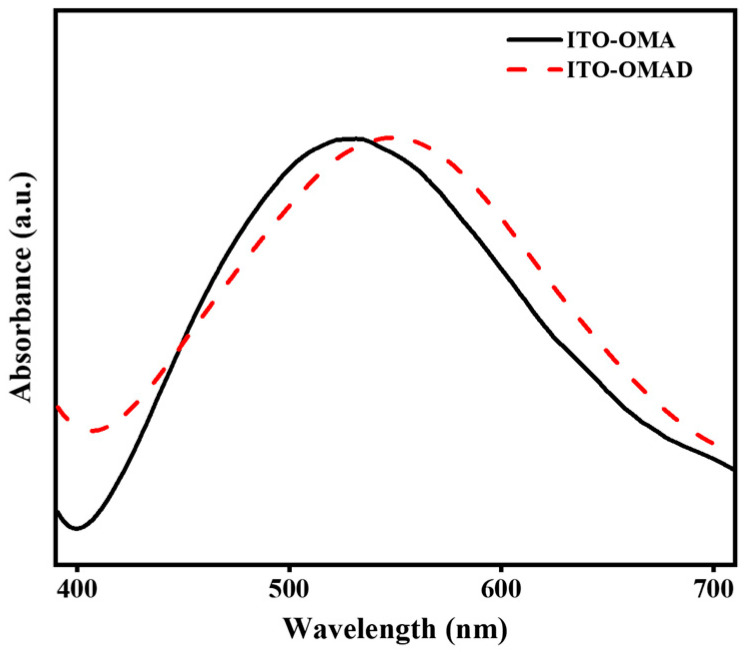
UV-Vis spectra of ITO-OMA electrode (black solid line) and ITO-OMAD electrode (red dashed line) normalized at the LSPR peak maxima.

Figure 5 shows the UV-Vis spectra of the ITO-OM electrode after immersion in a toluene solution of AuNPs (black solid line) and a toluene solution of AuNPs-DTP (red dashed line) for 18 h. It is evident that the wavelength of the LSPR absorption peak differs significantly between the ITO-OMA and ITO-OMAD electrodes, spanning a range of about 500–600 nm. Compared to the ITO-OMA electrode, the LSPR peak of the ITO-OMAD electrode is slightly red-shifted. These observations may be ascribed to the nitroxide pendants present on the surface of AuNPs, which can induce changes in the optical properties of AuNPs.

The reversible regulation of the optical properties of AuNPs is achieved by constructing an electrochemical switch (AuNPs-DTP) based on the as-prepared ITO-OMAD electrode. To verify the reversible regulation of the AuNP optical properties, the changes in the UV-Vis absorption peaks were measured before and after applying a voltage to the ITO-OMAD electrode. By applying a specific voltage to the ITO-OMAD electrode, the state of the nitroxide radicals is altered, leading to changes in the electronic and structural properties of the AuNPs. These changes manifest as shifts or variations in the UV-Vis absorption peaks, confirming the successful reversible modulation of the AuNP optical properties.

**Figure 6:**
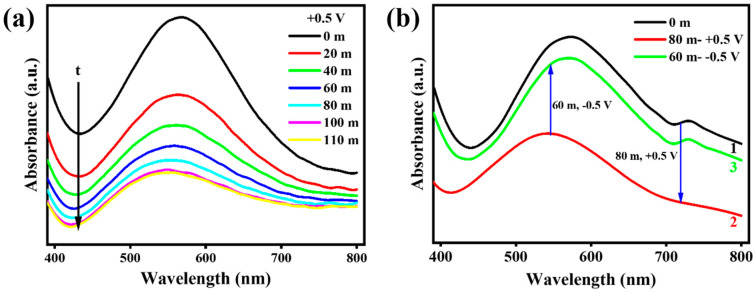
Electrochemical regulation of optical properties of AuNPs. (**a**) The LSPR variations of AuNPs while biasing +0.5 V on the ITO-OMAD electrodes with increasing time. (**b**) The attempt of the proposed electrochemically reversible switch: the initial UV-Vis spectrum is presented for the ITO-OMAD electrode in curve 1, followed by applying +0.5 V to the electrode for 80 min to obtain curve 2, and finally, applying −0.5 V to the electrode for 60 min to obtain curve 3.

As shown in Figure 6a, as the duration of the applied voltage (+0.5 V) increases, the LSPR absorption peaks of AuNPs in the range of 500–600 nm gradually undergo a blue shift, accompanied by a reduction in the adsorption intensity. Figure 6b reveals that, after applying a voltage of +0.5 V for 80 min to the ITO-OMAD electrode, the UV-Vis absorption peak of the AuNPs undergoes a blue shift and the absorption intensity decreases. Subsequently, upon applying a voltage of −0.5 V for 60 min [15], the UV-Vis absorption peak of AuNPs red-shifts back to the original position, and the absorption intensity is restored to approximately 80%. The possible reason for the recovery to 80% is Au-S bond cleavage, causing the loss of AuNPs after the voltage is applied. The findings presented above confirm the capability of the constructed electrochemical switch to achieve the reversible regulation of the optical properties of AuNPs.

**Figure 7:**
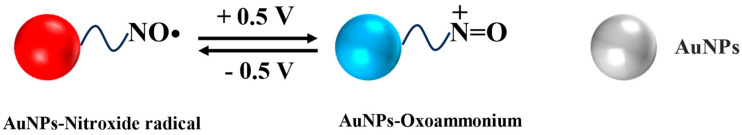
The proposed mechanism of the electrochemical regulation of the optical properties of AuNPs. Under the applied voltage of ±0.5V, the AuNP-DTP complex can reversibly switch between the two states of nitroxide radicals and oxoammonium, and then reversibly regulate the optical properties of AuNPs.

According to the findings of Gerken [44] et al., under neutral and alkaline conditions, TEMPO can easily reversibly form oxoammonium from nitroxide radicals. Therefore, the working mechanism of this electrochemical switch is caused by a reversible redox reaction between nitroxide radicals and oxoammonium (Figure 7); however, the current platform cannot achieve 100% recovery, as indicated by Figure 6b. In order to solve this problem, we propose two possible future plans: (1) binding AuNPs to the interface through more stable chemical bonds, for example, an amide reaction between carboxy-functionalized AuNPs and an amino-functionalized interface, or (2) adjusting the pH of the solution to allow the occurrence of an alternative redox reaction between N–O• and N–O, which will protect the Au-S bond.

## 3. Materials and Methods

### 3.1. General Materials

4-Hydroxy-2,2,6,6-tetramethylpiperidine-N-oxyl (4-OH-TEMPO, 99%), 1-ethyl-3-(3-dimethylaminopropyl) carbodiimide hydrochloride (EDCl, 99%), 4-dimethylaminopyridine (DMAP, 99%), 3,3′-dithiodipropionic acid (DTA, 99%), and 1-butanethiol (99%) were purchased from Adamas-beta. Tetrachloroauric acid trihydrate (HAuCl_4_·3H_2_O, Au 49–50%), ammonia (NH_3_·H_2_O, AR), and pentafluorophenylhydrazine (98%) were commercially available from Macklin. Potassium chloride (KCl, 98%), tetrabutylammonium hexafluorophosphate (TBAPF_6_, 98%), methyltrioctylammonium bromide (97%), acetonitrile (99%), and tetrahydrofuran (99%) were purchased from Aladdin. Hydrochloric acid (36–38%), nitric acid (95–98%), toluene (99.5%), ethanol (99%), hydrogen peroxide (H_2_O_2_, CR, 30%), acetone (AR), sodium borohydride (94%), potassium ferricyanide (K_3_[Fe(CN)_6_], 99%), and anhydrous sodium sulfate (99%) were purchased from Sinopharm Chemical Reagent. n-Hexane (AR), cyclohexane (AR), and ethyl acetate (AR) were purchased from Greagent. 3-Mercaptopropyltrimethoxysilane (MPS, 98%) was purchased from BD Medical. H_2_O was obtained from a Xiamen Ruisijie RODI water purification instrument. Rapid chromatography was performed on a high-quality 100–200 mesh silica gel from Macklin. Thin-layer chromatography was performed using a Qingdao Ocean thin-layer chromatography plate (0.25 nm thick).

### 3.2. Instrumentation and Methods

All electrochemical experiments were performed in a conventional one-component, three-electrode cell with the modified ITO electrode as the working electrode (15 mm × 50 mm), a platinum wire (1 mm) as the counter electrode, and a Ag/AgCl electrode (saturated KCl) as the reference electrode. The absorbance of the modified surfaces was measured using a UV-Vis spectrophotometer (UV-3600i Plus, SHIMADZU, Tokyo, Japan). The detailed microstructure morphology of AuNPs was observed through a high-resolution transmission electron microscope (HRTEM, JEOL, Tokyo, Japan). The morphology of the ITO electrode surface was examined using a scanning electron microscope (JSM-7800F, TESCAN, Brno, Czech Republic). The composition and molecular weight of the synthesized TEMPO derivatives were analyzed using a superconducting nuclear magnetic resonance spectrometer (NMR, AVANCE II 400, Bruker, Billerica, MA, USA) and liquid chromatography with ion trap mass spectrometry (LC/MS, Agilent, Palo Alto, California, USA), respectively. The nitroxide radical structure of the synthesized TEMPO derivatives was analyzed using electron spin resonance spectroscopy (ESR, A300-10/12, Bruker, Billerica, MA, USA).

### 3.3. Material Synthesis

**Scheme 1:**
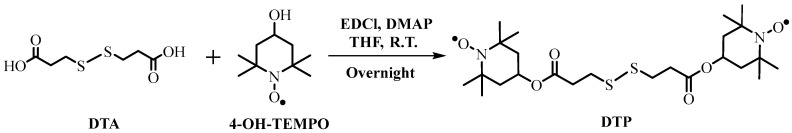
Synthesis route of DTP via the esterification between DTA and 4-OH-TEMPO.

*Synthesis of bis [2-(4-oxy-2,2,6,6-tetramethylpiperidine 1-oxyl)ethyl] disulfide, DTP* [39,45]: As displayed in Scheme 1, DTA (0.631 g, 3 mmol), 4-OH-TEMPO (1.55 g, 9 mmol), EDCl (1.73 g, 90 mmol), and DMAP (0.22 g, 18 mmol) were added to a 100 mL round-bottom flask and purged with dry N_2_ for 15 min. Subsequently, anhydrous tetrahydrofuran (25 mL) was added, and the mixture was stirred at room temperature for 12 h. After the completion of the reaction, the white precipitate was filtered using a Brinell funnel, and the remaining red oil was obtained by rotary evaporation to remove the solvent. A thin-layer chromatography plate (cyclohexane/ethyl acetate, 5:5) was used for the initial analysis, revealing the presence of both the desired product (DTP) and an unreacted substrate, 4-OH-TEMPO. To further separate the reaction products, the mixture was then subjected to rapid chromatography, employing a column packed with silica gel, and the volume ratio of cyclohexane/ethyl acetate was gradually changed from 8/2 to 5/5. The resulting orange-red oil was obtained by rotary evaporation, and was subsequently recrystallized with n-hexane to give an orange solid (0.826 g, 1.59 nmol). ^1^H NMR (300 MHz, CDCl_3_): δ (ppm) = 5.17–5.02 (m, 2H), 2.93 (m, 4H), 2.73 (m, 4H), 1.93 (m, 4H), 1.58 (m, 4H), and 1.40–0.99 (m, 24H). MS (DTP, *m*/*z*): [M+H]^+^ calculated as 518.25 and found to be 519.25.

*Synthesis of AuNPs*: AuNPs were synthesized using the improved Brust–Schiffrin method [40,46]. First, a solution of HAuCl_4_·3H_2_O (1 mL, 250 mM) and a toluene solution of methyltrioctylammonium bromide (100 mL, 20 mM) were vigorously mixed and stirred until the complete transfer of AuCl_4^−^_ ions to the oil phase was achieved. Next, 0.5 mmol of 1-butanethiol was added with vigorous stirring, resulting in a rapid color change from wine red to clear and transparent. When the solution temperature dropped to between 0 and 15 °C, a freshly prepared solution of sodium borohydride (5 mL, 0.1 M) was quickly added and the mixture was stirred vigorously. After stirring for 3 h at 15 °C, the water phase was removed, and the oil phase was washed with pure water (50 mL × 2) before being concentrated to 2 mL via spinning. The crude product was mixed with 100 mL of anhydrous ethanol and stored at −18 °C for 4 h. Subsequently, ultrasonication for 60 s was performed, followed by centrifugation (5 min, 10,000 rpm) to collect the precipitate. The precipitate was then dissolved in a toluene solution (2 mL), subjected to centrifugation with EtOH (50 mL × 2), and washed with n-hexane (50 mL × 10). Finally, the obtained AuNPs were vacuum dried at 60 °C.

Synthesis of AuNPs-DTP [40]: 5 mg of AuNPs and 5 mg of DTP were mixed in 5 mL of toluene for 72 h, and the resulting mixture was evaporated to obtain a black solid. Then, 0.5 mL of toluene was added to dissolve the solid, followed by the addition of 20 mL of acetone to allow the precipitation of the synthesized AuNPs-DPT. The mixture was then stored at −18 °C for 2 h before undergoing centrifugation (5 min, 10,000 rpm). The precipitate obtained was further centrifuged and washed with acetone (20 mL) until no trace of excessive DTP was detected by thin-layer chromatography.

### 3.4. Pretreatment of ITO Electrode

**Scheme 2:**
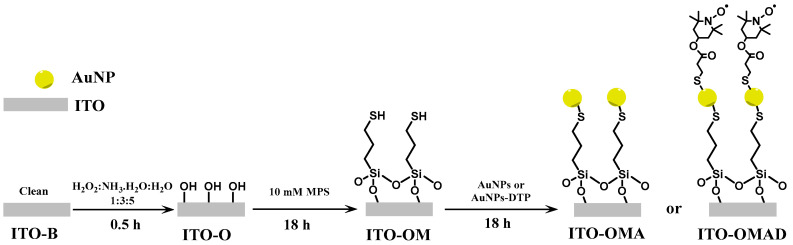
Schematic representation of constructing SAM on the surface of the ITO electrode.

The ITO electrode underwent a series of ultrasonic cleaning steps with toluene, acetone, ethanol, and ultrapure water, each lasting for 15 min. The electrode was then dried under a stream of N_2_ gas. The resulting clean ITO electrode was named ITO-B. Subsequently, the ITO-B electrode was immersed in a mixture of hydrogen peroxide, ammonia, and water (H_2_O_2_:NH_3_·H_2_O:H_2_O = 1:3:5) for a duration of 30 min [43]. After the reaction, the electrode was thoroughly rinsed with ultrapure water several times and dried under N_2_. The pretreated ITO electrode was labeled as ITO-O. A simple illustration of the process is shown in Scheme 2.

### 3.5. Preparation of Self-Assembly Monolayers (SAM) on Pretreated ITO Electrode

The synthesis and configuration of the electrodes are also displayed in Scheme 2. The ITO-O electrode was immersed in a toluene solution of 10 mM MPS for 18 h, followed by washing 2–3 times with toluene and ultrapure water and drying under a N_2_ atmosphere. The prepared ITO-O electrode modified by MPS was designated as ITO-OM [43].

After confirming the successful formation of a high-quality SAM of MPS on the ITO-O electrode through an electrochemical analysis, the ITO-OM electrode was immersed in a toluene solution of 0.2 mg/mL of AuNPs-DTP for 18 h. Subsequently, the electrode was washed 2–3 times with toluene and ultrapure water and dried with N_2_ gas. The forming electrode modified with AuNPs-DTP was denoted as ITO-OMAD. In order to investigate the effect of nitroxide radicals on the optical properties of AuNPs, a control electrode named ITO-OMA was prepared; in particular, the ITO-OM electrode was immersed in a toluene solution of 0.2 mg/mL of AuNPs for 18 h. After washing with toluene and ultrapure water 2–3 times, the control electrode was dried under N_2_ gas.

Finally, the two electrodes, ITO-OM and ITO-OMAD, were selected for an electrochemical analysis and the characterization of their surface morphology. Additionally, the ITO-OMAD electrode was employed to examine the influence of electrochemical regulation on the optical properties of AuNPs.

## 4. Conclusions

In summary, this study successfully introduced a novel approach for regulating the optical properties of AuNPs. By utilizing nitroxide radicals to modify AuNPs and by constructing an electrochemical reversible switch, the open/closed shell state of nitroxide radicals can be altered through electrochemical oxidation and reduction, enabling the reversible regulation of the absorption intensity of AuNPs. The experimental results demonstrate that the optical properties of AuNPs can be effectively and reversibly regulated by applying an external voltage of ±0.5 V when using the ITO-OMAD electrode as the working electrode. This approach offers simplicity in its operation and convenience in detection compared to existing methods for regulating the photophysical properties of AuNPs while ensuring electrochemical reversibility. Additionally, the SAM platform constructed by AuNPs-DTP on the MPS-modified ITO was analyzed using CV technology. This platform can be used as a medium for electron transfer to study the photoelectric properties of AuNPs on the surface. However, the stability of the electrochemically reversible regulation of the optical properties of AuNPs requires further elucidation; for example, the possibility of attaching AuNPs to the electrode substrate via more robust chemical bonds should be investigated. As the field of the electrochemically reversible regulation of the optical properties of AuNPs continues to advance, this proof-of-concept is expected to become a new research hotspot in the future, offering promising possibilities for the application of other nanomaterials.

## Data Availability

Not applicable.
